# Stem Surface Microstructure and Plant–Insect Interactions: A Perspective

**DOI:** 10.3390/plants15182751

**Published:** 2026-09-09

**Authors:** Elena V. Gorb, Stanislav N. Gorb

**Affiliations:** Department of Functional Morphology and Biomechanics, Zoological Institute, Kiel University, Am Botanischen Garten 1–9, 24118 Kiel, Germany; sgorb@zoologie.uni-kiel.de

**Keywords:** ant, attachment, biomechanics, epicuticular wax, mechanical filter, plant surface, pubescence, trichome, attachment

## Abstract

Plant surfaces form a critical biomechanical and ecological interface between plants and insects. Beyond chemical cues and rewards, the physical micro- and nanostructure of stems can decisively shape insect attachment, locomotion, and visitation patterns. This perspective paper synthesizes a body of work centered on studies by Gorb, Gorb, and collaborators (2017–2024), together with the broader literature cited therein, to develop a framework for understanding stem surface-mediated plant–insect interactions. We integrate comparative, experimental, and biomechanical evidence demonstrating that epicuticular waxes, pubescence, epidermal cell geometry, and surface roughness act each and in combination as a mechanical filter for crawling insects, particularly ants. We propose a unifying conceptual model, in which plant stem surfaces function as scale-dependent mechanical barriers that modulate insect access, balancing ecological trade-offs between attraction and exclusion. The paper further highlights ecological and evolutionary implications as well as methodological advances and discusses opportunities for bioinspired applications.

## 1. Introduction

Plant–insect interactions have traditionally been interpreted primarily through chemical ecology, nutritional rewards, and trophic relationships. Defensive compounds, volatile cues, and mutualistic exchanges have dominated conceptual frameworks explaining how plants regulate insect access and behavior [1]. However, plants are also physical objects, and insects must interact mechanically with plant surfaces before and during any chemical interaction can take place [2]. For crawling insects in particular, the ability to attach to and move along plant stems represents a fundamental prerequisite for exploiting plant-associated resources.

In recent decades, research in functional morphology and biomechanics has increasingly demonstrated that the physical micro- and nanostructure of plant surfaces can exert strong control over insect attachment and locomotion as well as behavioral decisions related to those. Stem surfaces may act either as conduits facilitating insect movement or as barriers that effectively exclude certain taxa [3,4,5]. These effects are often subtle at the structural level but profound in their ecological consequences.

To attach themselves to a variety of substrates, insects have evolved two distinctly different types of adhesive structures (pads): smooth pads and fibrillar (hairy, setose) ones [6]. Recent phylogenetic analyses of pad characteristics, conducted alongside traits from other organ systems, have helped to clarify pad evolution and indicated that these structures have evolved independently multiple times [7]. Such groups as orthopterans, phasmatodeans, hymenopterans, and thysanopterans bear smooth pads, whereas dipterans, coleopterans, and dermapterans possess mainly fibrillar pads. In coleopterans, hymenopterans (where ants belong), heteropterans, phasmatodeans, and mantophasmatodeans, both types of attachment pads have been reported. Adhesive structures are not restricted to one particular region of the leg. For example, they may be located on claws, derivatives of the pretarsus, tarsal apex, tarsomeres, or tibia. Fibrillar systems always have cuticle protuberances on their surfaces. Adhesive setae and acanthae are hollow inside and rich in porous channels. In some dipterans, acanthae may additionally contain pores under the terminal plate. Flexibility of the material in the smooth attachment pads due to its foam-like or fiber-like internal structure or compliance of the arrays of fine surface outgrowths in the fibrillar pads leads to the maximization of the possible contact area with a wide range of substrate profiles, including smooth ones [8,9].

Since ants are numerous, omnipresent, live in colonies, and interact a lot with plants, a growing body of experimental and comparative studies has focused on the interaction between plant stem surfaces and ants [3,4,5,10,11]. Ants are ideal model organisms in this context because they are abundant, behaviorally flexible, and, being wingless, rely heavily on surface attachment for locomotion. Moreover, ants possess a wide spectrum of ecological roles [12], ranging from mutualistic defenders [3,5,13] to exploiters and pollination antagonists [14,15,16,17,18,19,20]. As such, mechanisms that regulate ant access to plant surfaces can potentially have cascading effects on plant fitness.

This perspective paper is built upon a series of studies by Gorb, Gorb, and collaborators (2011–2024), while substantially expanding the scope to include the broader literature cited in these works and related foundational studies. In the present paper, by integrating biomechanics, surface functional morphology, behavioral ecology, and evolutionary perspectives, we aim to develop a comprehensive framework that explains how stem surface microstructure can function as a mechanical filter in plant–insect interactions.

Additionally, this perspective paper situates the phenomenon of mechanical filtering within a general theory of plant defense, linking microstructural variation to ecological and evolutionary processes. We aim to identify general principles, highlight gaps in current understanding, and propose some avenues for future research that integrate experimental, comparative, and theoretical approaches.

## 2. Mechanical Filtering at the Plant Surface

### 2.1. The Plant Surface as a Mechanical Filter

**Figure 1:**
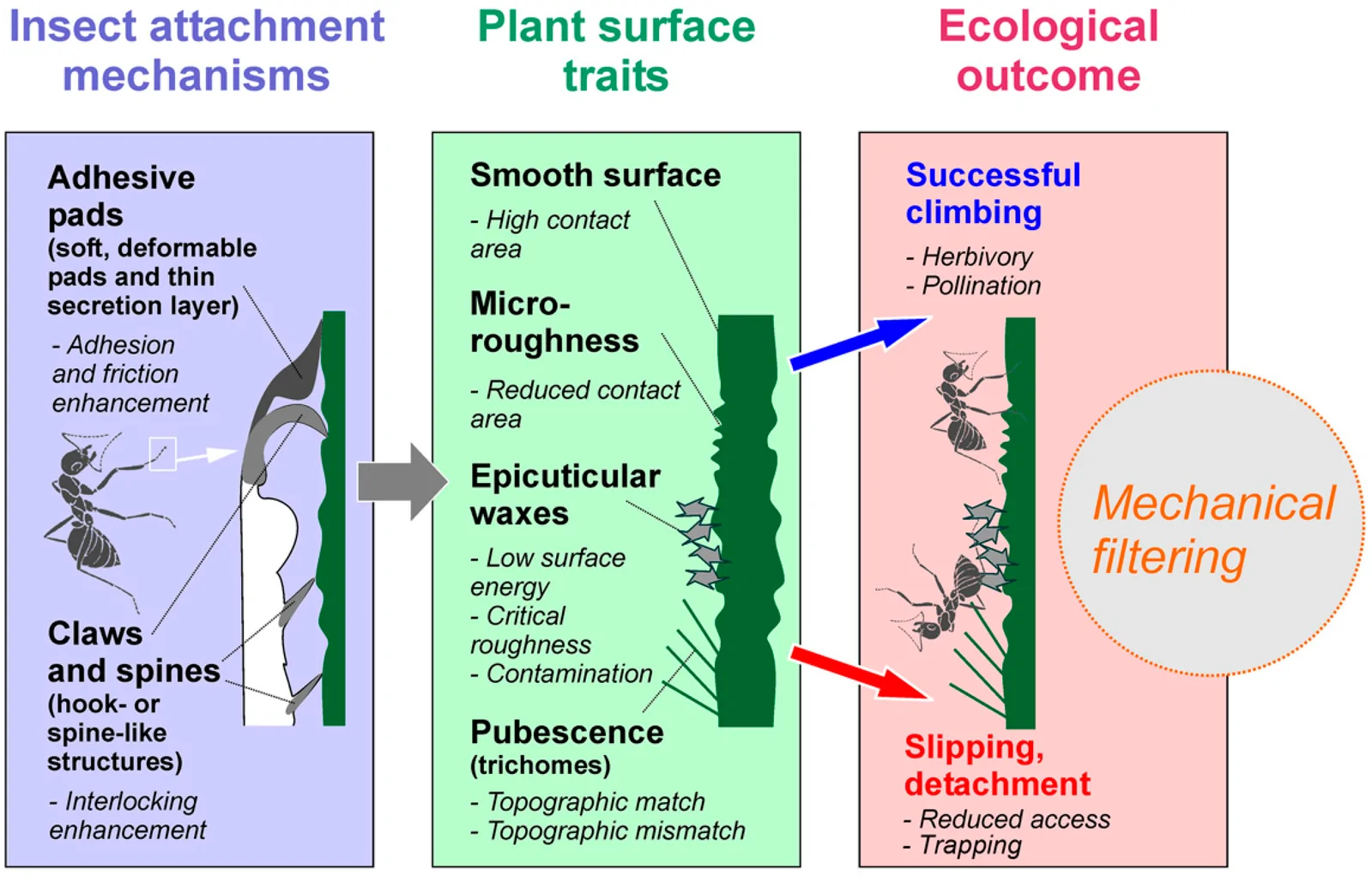
Conceptual framework of mechanical filtering. Schematic illustrating how plant stem surface traits, including epicuticular waxes, pubescence, and micro-roughness, interact with insect attachment mechanisms such as adhesive pads and claws/spines. Depending on the mechanical compatibility between the plant surface and insect attachment system, insects may either successfully climb or slip and/or avoid the surface. Thereby, access of different insect species can be regulated to some extent. This influences ecological interactions and plant fitness through mechanical filtering.

We use the term *mechanical filtering* to describe *the process by which physical characteristics of a plant surface differentially modify the probability that insects with particular attachment and locomotory capabilities can access, traverse, and remain on that surface.* Mechanical filtering therefore emerges from the interaction between plant surface traits and insect attachment systems, rather than from the presence of any particular plant surface structure alone. Surface characteristics such as epicuticular waxes, micro-roughness, epidermal cell geometry, and trichomes can interfere with attachment through different mechanisms, including reduction of effective contact area, scale mismatch between plant surface asperities and insect attachment structures, claw-interlocking failure, contamination of adhesive pads, entanglement, and changes in friction or lubrication (Figure 1). These biomechanical effects can subsequently alter insect climbing success, locomotory stability, and visitation behavior.

Mechanical filtering is therefore a functional and ecological concept, rather than a synonym for a slippery or anti-adhesive plant surface. A slippery surface or an anti-adhesive cuticle describes a physical property or biomechanical effect that may contribute to filtering, whereas mechanical filtering refers to the resulting differential effect on insect access. A given surface trait constitutes a mechanical filter when its physical properties interact with the attachment and locomotory characteristics of insects in such a way that some insects are more strongly impeded than others, or when access probability is progressively reduced across insects differing in attachment ability. Thus, wax bloom alone may contribute to mechanical filtering if it reduces access or climbing success in a trait-dependent manner, whereas a multi-trait configuration, such as the combination of three-dimensional waxes, pubescence, and surface roughness, may generate a stronger filtering effect.

Mechanical filtering should consequently be viewed as a continuum ranging from relatively weak reduction in attachment performance or visitation to strong and, in some cases, near-complete exclusion. The important distinction is that filtering concerns the relationship between surface properties and the distribution of insect performance or access, rather than the absolute slipperiness or adhesiveness of the surface itself. In this framework, the “greasy pole syndrome” and similar stem surface syndromes represent particular examples of mechanical filtering, in which several surface traits act simultaneously or synergistically to reduce access by particular insect groups.

### 2.2. Scale-Dependent Interactions and Attachment Failure

**Figure 2:**
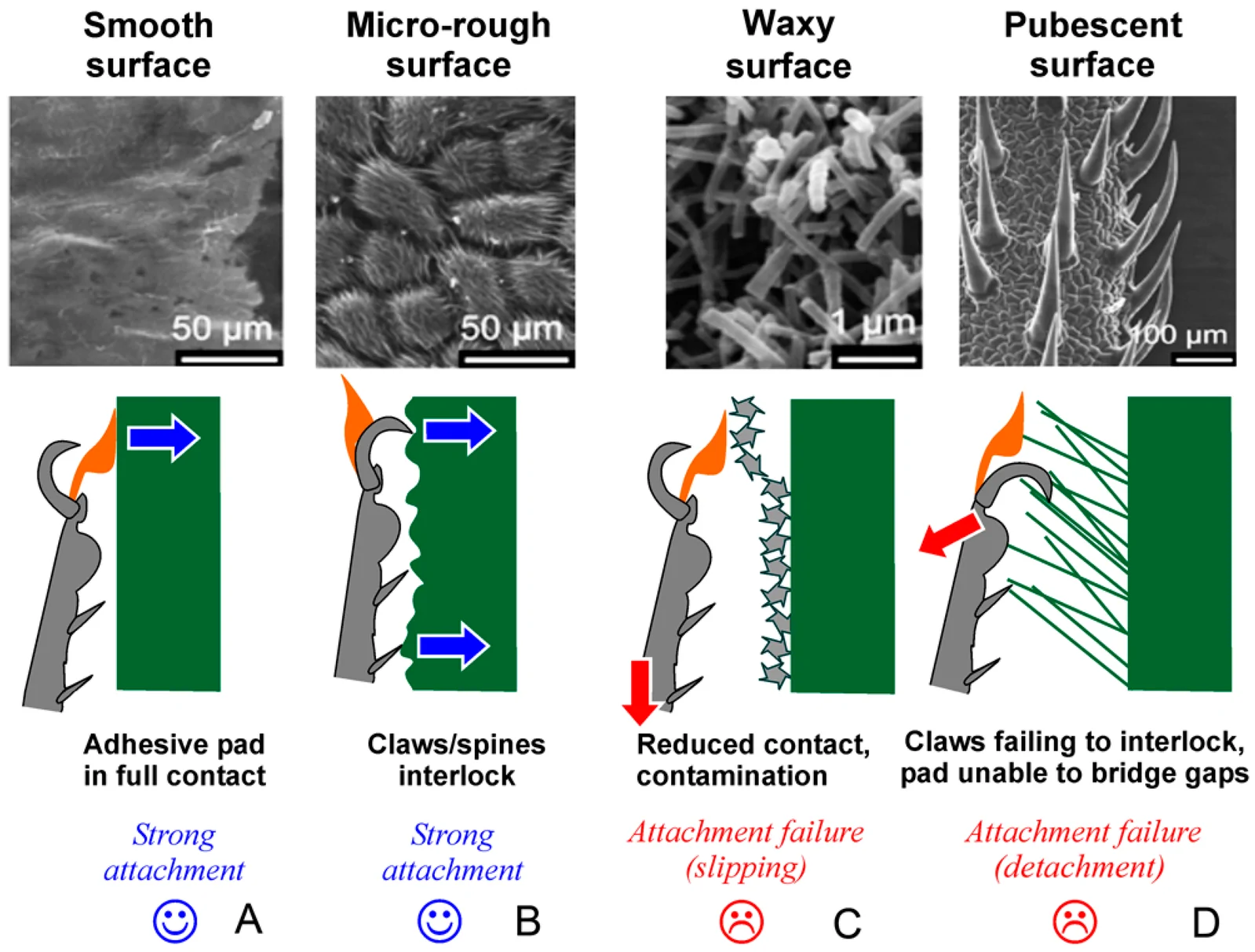
Comparison of stem surfaces (scanning electron microscopy, SEM) and ant attachment outcomes. Representative scanning electron micrographs of plant surfaces showing (**A**) smooth cuticle (*Pyrus communis* L. (Rosaceae)), (**B**) micro-rough surface (*Syringa vulgaris* L. (Oleaceae)), (**C**) 3D epicuticular wax (*Chelidonium majus* L. (Papaveraceae)), and (**D**) dense trichome coverage (*Geranium sanguineum* L. (Geraniaceae)). Insets schematically illustrate insect attachment responses to each surface type. Smooth wax films/layers generally permit extensive pad contact and relatively high attachment performance, whereas structured wax layers can reduce attachment performance through several mechanisms, including reduced effective contact area and pad contamination. Micro-rough surfaces, depending on their dimension, might support claw or/and spine interlocking. Dense trichome layers create strong topographic mismatches that prevent effective pad contact and claw interlocking. Blue arrows in (**A**,**B**) show where the pressure is applied. Red arrows in (**C**,**D**) indicate the shear force applied. SEM images are adapted from Ref. [23].

A central concept emerging from the literature is scale matching between plant surface asperities and insect attachment structures. Insects employ claws, adhesive pads, or combinations thereof (Figure 1) [6]. When surface roughness falls outside the effective size range for claw interlocking or pad conformity, attachment performance may decrease, with the magnitude of the effect depending on the attachment system and the characteristics of the surface. Plant epicuticular wax projections, micro-rough cuticular folds, and dense trichomes can all generate such mismatches, leading to slipping, pad contamination, and therefore to an unstable gait (Figure 2) [21,22]. This scale-dependent mismatch has been demonstrated experimentally using ants, beetles, and other small arthropods [3,23,24,25,26,27]. In this context, we do not define “strong attachment” by a fixed percentage of insects adhering to the surface. Instead, attachment strength is considered relative to an appropriate smooth or otherwise suitable control surface and to the specific experimental measure used. Strong attachment refers to attachment performance that is comparable to that of the control under the experimental conditions, as assessed by the relevant quantitative or behavioral measure (e.g., adhesive/frictional force, climbing success, locomotory performance, or frequency of slipping). “Reduced attachment” means that these measures are significantly lower than in the control. The term “attachment failure” is used for cases in which reduced attachment performance results in a clear functional failure, such as slipping, detachment, falling, or inability to traverse the experimental surface. Thus, attachment failure is not defined simply by the absence of a statistically significant difference or by a particular percentage of insects failing to attach.

### 2.3. The Greasy Pole Syndrome as an Emergent Property

**Figure 3:**
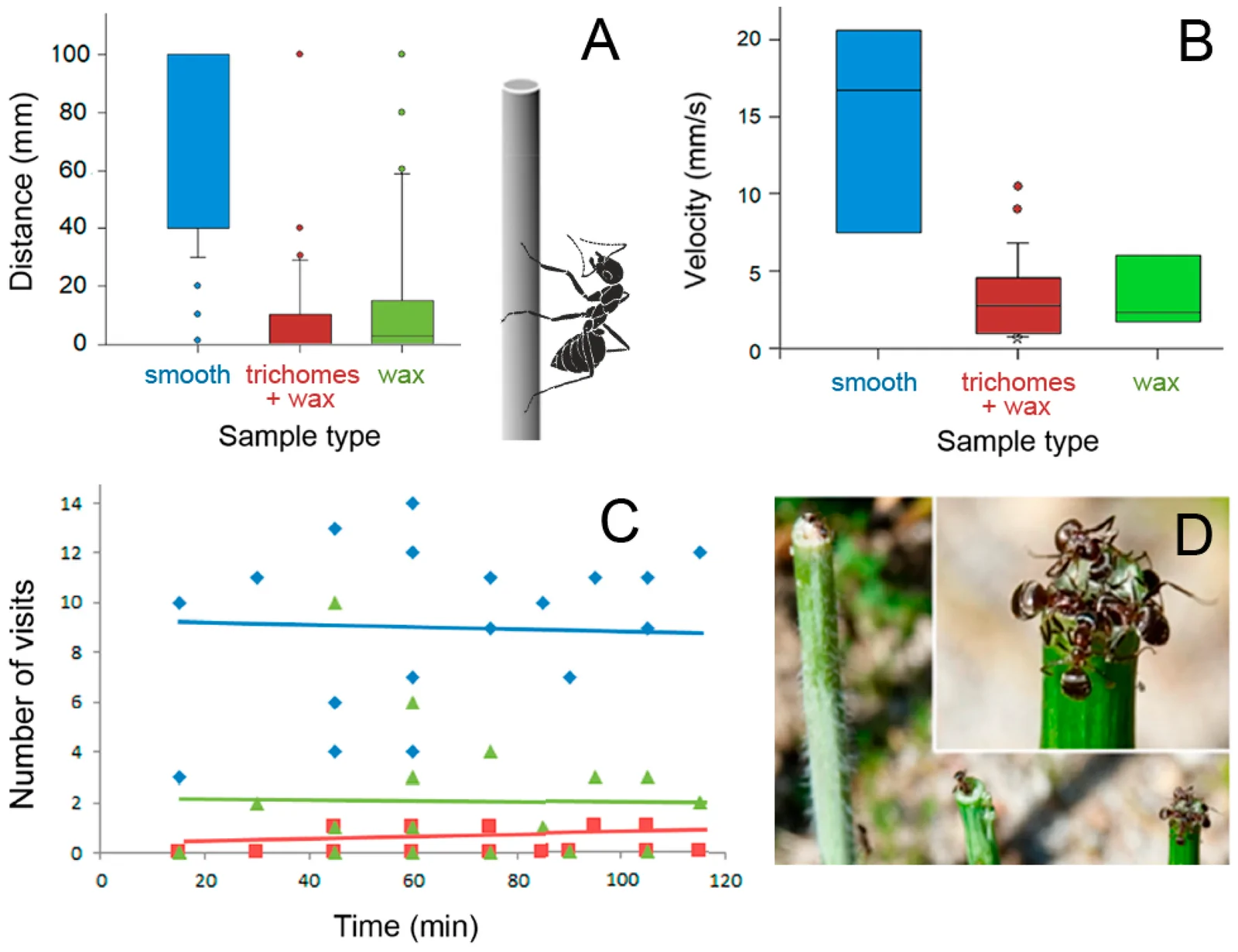
Behavioral response illustrating reduction **of** locomotory activity in ants. Attachment performance (panels (**A**,**B**)) is shown for three representative plant stem surfaces of *Alliaria petiolata*: smooth surface (blue), with 3D wax (green), and with additional dense trichome coverage (red). Maintaining stable contact on smooth stem samples allows ants to have higher attachment performance (i.e., significantly longer traversed distances (**A**) and higher running velocities (**B**)), while reduced effective adhesion causes lower performance. Panels (**C**,**D**) show the corresponding visitation frequencies of ants on these surfaces (the number of ants recorded on each stem sample type during the experimental time (**C**) and ants visiting different tested stem samples (**D**)), highlighting a positive correlation between attachment performance and likelihood of successful climbing or exploration. Surfaces incompatible with ant attachment (3D wax, dense pubescence) exhibit reduced visitation, demonstrating the functional consequences of mechanical filtering at the plant–insect interface. Lines and symbols in (**C**) represent linear regressions and experimental results, respectively, on the smooth stem surface (blue, rhombuses), stems with 3D wax (green, triangles) and additional dense trichome coverage (red, squares). Adapted from Ref. [28].

Described initially in plants from the genera *Salix* (Salicaceae), *Hypenia,* and *Eriope* (both Lamiaceae) [10,11] and experimentally studied recently in *Alliaria petiolata* (M. Bieb.) Cavara & Grande (Brassicaceae) [28], the “greasy pole syndrome”, hindering the access of ants to terminally located reproductive plant organs and, by this, preventing ants from robbing flower nectar and other resources, exemplifies mechanical filtering in action. Here, three-dimensional (3D) epicuticular wax coverage and pubescence jointly reduce ant climbing success, not through a single dominant trait, but via synergistic interference with multiple attachment mechanisms (Figure 3). Compared to control, mechanically wiped stem samples lacking any surface features (Figure 3D inset) and even to waxy stems, hairy + waxy plant stems were less often visited by generalist ants *Lasius niger* (Linnaeus, 1758) (Insecta, Hymenoptera, Formicidae) (Figure 3C,D), where they traversed significantly (almost ten-fold) shorter distances (Figure 3A) and moved with significantly (up to seven-fold) lower velocities compared to the smooth samples (Figure 3B). Similar effects have been documented in woody bamboo species (“stem guard syndrome” hampering the access of ants to aphid colonies located on young culms due to reducing ant attachment ability to culm surfaces) and some herbaceous plants, e.g., *Alchemilla mollis* (Buser) Rothm. (Rosaceae), *Lilium lancifolium* Thunb. (Liliaceae), *Salvia nemorosa* L. (Lamiaceae), *Smyrnium rotundifolium* Mill. (Apiaceae), where combinations of roughness, wax crystals, and trichomes reduce access for non-mutualistic insects [24,25,29,30]. These multi-trait effects underscore the importance of considering mechanical filtering as a system-level property rather than a collection of independent traits.

### 2.4. Integration with Ecological Theory

Mechanical filtering can be conceptualized within broader frameworks of plant defense, including the growth–differentiation balance hypothesis [31,32,33] and the optimal defense theory [34,35,36]. In these models, plants allocate resources to defenses according to ecological risk and developmental stage. Surface traits that reduce insect access without imposing substantial metabolic costs may represent evolutionarily stable strategies, particularly in plants interacting with a mix of both beneficial and antagonistic insects. However, these theories need more experimental evidence: growth–differentiation balance and optimal defense are invoked, but there are limited data in the literature (e.g., actual costs of wax vs. trichome production, measured fitness trade-offs along stems or among organs). Similarly, the claim that surface traits allow plants to fine-tune ant access to reproductive structures is plausible, but most of the evidence present is still correlative. In light of the above, the mechanoecology of insect–plant interactions is a very important future direction of research, which was largely overlooked previously (reviewed in Ref. [37]).

## 3. Stem Surface Microstructure: Diversity and Functional Consequences

### 3.1. Epicuticular Waxes

Epicuticular waxes occur as smooth films/layers or 3D projections. 3D waxes covering different plant organs are particularly effective at reducing insect attachment (adhesion and friction) (Figure 2C) (reviewed in Refs. [22,38,39,40]). Such an impact has been revealed in a large number of plant and insect species using direct behavioral observations as well as various experimental approaches, starting from rather simple inversion/incline tests [23,41] up to precise force measurements by applying, for example, pulling and centrifugal techniques [4,42]. In addition, not only the presence of 3D wax structures, but also their size, distribution, and abundance affect insect attachment [43].

**Figure 4:**
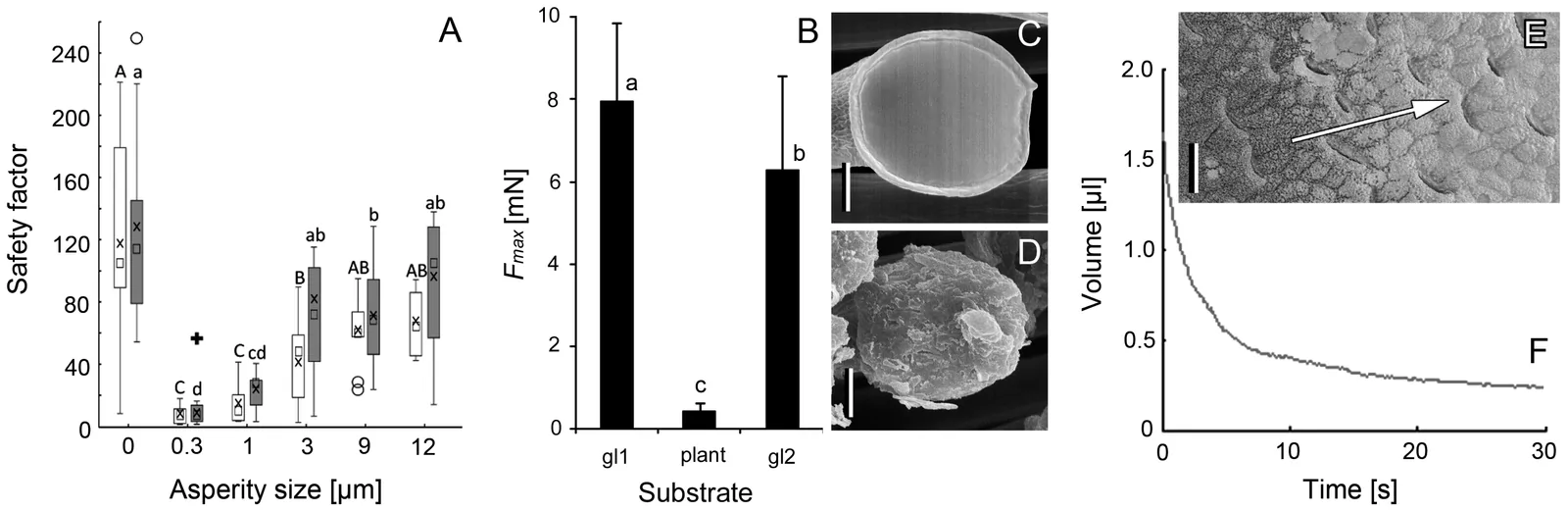
Results of the exemplary experiments testing the roughness hypothesis (**A**), contamination hypothesis (**B**–**D**), and (3) fluid absorption hypothesis (**E**,**F**). (**A**) Safety factor (force divided by insect weight) for the attachment (traction) force of female ladybird beetles *Propylea quatuordecimpunctata* (Linnaeus, 1758) (Insecta, Coleoptera, Coccinellidae) (white bars) and *Harmonia axyridis* (Pallas, 1773) (Insecta, Coleoptera, Coccinellidae) (gray bars) pulling on plastic surfaces having different micro-roughness (0, 0.3, 1, 3, 9, and 12 μm). Boxplots with different upper and lower case letters, respectively, are significantly different. In both insect species, significant multi-fold reduction was observed on 0.3 and 1 μm rough surfaces. (**B**) The maximum traction force *F_max_* obtained on waxy plant surfaces (plant) and in the first (gl1) and second (gl2) experiments on glass. Bars with different letters are significantly different. The force was significantly lower (ten-fold lower) in cases of all tested waxy surfaces compared to the reference experiment on glass gl1. After beetles had walked on waxy plant substrates, their adhesive pads were contaminated with wax material, and they demonstrated significantly lower force values in the gl2 test than in the gl1 test. (**C**,**D**) SEM micrographs of the setal tip of *Chrysolina fastuosa* (Scopoli, 1763) (Insecta, Coleoptera, Chrysomelidae) male beetles in clean condition (**C**) and after the beetle had walked on *Acer negundo* waxy stem surface (**D**). Note the contamination by wax material in (**D**). (**E**) Cryo SEM images of the wax coverage in *Nepenthes alata* Blanco (Nepenthaceae) pitcher after applying oil drops onto the pitcher surface. Arrow indicates the gradient from uncovered (dark) to completely wetted/oil-adsorbing (bright) regions. (**F**) Time-dependent values of the volume of the oil drop on the *N. alata* waxy surface. Scale bars: 1 μm (**C**,**D**) and 100 μm (**E**). Adapted from Ref. [48] (**A**), ref. [55] (**B**–**D**), and [60] (**E**,**F**).

Reduced insect attachment on waxy plant surfaces has been explained by the contribution of (i) specific surface micro-roughness (roughness hypothesis), (ii) contamination of insect adhesive pads by plant wax (contamination hypothesis), (iii) absorption of pad secretion by the wax coverage (fluid absorption hypothesis), and (iv) hydroplaning induced by dissolved wax (wax dissolution hypothesis) [23]. To date, the first three hypotheses were proven using experiments with either plant (mostly leaf) surfaces or artificial samples. A great reduction in the insect attachment force was measured on micro/nanorough surfaces, where asperity sizes corresponded to those of typical plant wax projections—0.3 and 1 μm (Figure 4A) [6,44,45,46,47,48]. Numerous microscopical, experimental, and theoretical studies confirmed contamination of insect adhesive organs by plant wax material (Figure 4C,D) and its diminishing effect on attachment (Figure 4B) [23,41,49,50,51,52,53,54,55,56,57,58,59]. The ability of the plant wax coverage to absorb oil, which is one (in beetles possibly even the main) component of the insect pad secretion, was demonstrated experimentally (Figure 4E,F) [60], and the impact of this capability on reduction of insect attachment was revealed in experiments with porous artificial substrates [61,62].

In experimental assays with plant stems, generalist ants exhibited reduced climbing success on waxy stems and visitation frequency correspondingly declined [24,25]. For example, in the study with completely and partly wiped waxy stems of *Anethum graveolens* L. (Apiaceae), *Dahlia pinnata* Cav. (Asteraceae), and *Tagetes patula* L. (Asteraceae), half of the tested ant individuals of *Lasius niger* refused at first to step on waxy surfaces, whereas others traversed significantly (up to five- or six-fold) shorter distances on these surfaces [24]. In cases of wax-bearing stems in *Alchemilla mollis* and *Tulipa gesneriana*, the number of visiting ants was drastically (more than ten-fold) reduced in comparison to smooth or hairy stems of other tested plant species, such as *Lilium lancifolium*, *Salvia nemorosa*, and *Paeonia lactiflora* Pall. (Paeoniaceae) [25].

Interestingly, a cursory glance at 343 higher plant species discovered wax-bearing stems in 70% of examined taxa, among which 24% had wax-free leaves [63]. The chemical composition and crystal morphology of epicuticular waxes are highly variable across taxa, providing a flexible toolkit for modulating insect interactions [64,65,66,67,68].

### 3.2. Pubescence and Trichomes

Trichomes can function as physical barriers that elevate insects away from the plant cuticle or restrict movement through entanglement (Figure 2D) [69]. This effect has been reported for herbivorous arthropods from various families, such as Chrysomelidae, Aphididae, Aleurodidae, Cicadellidae, Sphingidae, Noctuidae, and Tetranychidae. Shape, dimensions, spatial arrangement, abundance, the presence of secretion, as well as stiffness of trichomes determine their functional impact [70]. For example, densely situated and longer trichomes are generally more effective at preventing insect attachment. In particular, tight pubescence has been shown to reduce ant and beetle locomotion and also to interact synergistically with wax layers (Figure 3A,B) [25,27,28]. In general, trichome coverage negatively affects host-driven herbivore success [2], but may also provide a kind of refuge for tiny or specialized insect species via deterring, hampering, or even hurting their natural enemies, such as predatory or parasitoid insects (e.g., [71,72]). Morphological diversity in trichome architecture has been observed across angiosperms and gymnosperms, suggesting convergent evolution driven by insect interactions.

### 3.3. Epidermal Cell Geometry and Roughness

The shape and arrangement of epidermal cells may influence coarse cuticle surface roughness and, consequently, attachment. It is plausible to suppose that papillate or highly convex cells reduce the real contact area available for insect adhesive attachment organs, whereas smooth, flat cells facilitate attachment (Figure 2A). Reduced attachment on coarsely uneven plant surfaces was detected in a few previous studies [23,73,74], while a number of previous publications have reported either non-significant or even positive effects of the cell shape structuring on insect performance, especially on petal surfaces [26,75,76]. In many traps of carnivorous plants and kettle trap flowers, prominent downward-pointed cells/trichomes contribute to insect capture by reducing their grip to epidermal surfaces [26,77,78,79,80,81,82,83]. Comparative work on bamboo stems illustrates how subtle differences in epidermal geometry translate into marked differences in insect attachment performance [30]. Micro-rough plant cuticles including those ornamented with cuticular folds, often in combination with waxes or trichomes, have been shown to generate complex topographies that impede insect movement in a taxonomically generalizable way [21,75,81,84,85].

## 4. Insect Attachment Mechanisms and Behavioral Responses

### 4.1. Biomechanics of Ant Attachment

Ants such as *Lasius niger* employ a combination of claws and adhesive pads to traverse diverse substrates. Attachment success depends on a combination of frictional forces, pad adhesion, and mechanical interlocking between claws and surface asperities [86,87]. The mechanical properties of the tarsal pads, including secretion viscosity and pad compliance, determine how effectively ants can navigate smooth, waxy, or (micro-)rough surfaces. Loss of adhesion as well as slipping and missteps are commonly observed on stems with incompatible microstructures, supporting the mechanical filtering concept.

Experimental studies have shown that ants are sensitive to nanoscale roughness and surface contamination. Even small quantities of detached wax crystals can reduce adhesive contact and increase slipping events [23,47]. This mechanical sensitivity explains why seemingly minor differences in epidermal microtopography or wax coverage result in pronounced differences in ant climbing success and visitation frequency (Figure 3C) [24,25,30].

### 4.2. Behavioral Consequences of Surface Properties

Behavioral assays demonstrate that ants avoid surfaces that compromise attachment or increase the risk of slipping. The reduction in visitation frequency (Figure 3A) is thus a combination of immediate locomotion failure and longer-term learned avoidance. These effects have been quantified experimentally using choice assays and artificial stems with controlled surface treatments [24,28,29]. Observations indicate that ants may preferentially select stems that provide adequate grip, even when these surfaces offer lower nutritional rewards, highlighting the functional importance of mechanical cues in decision-making.

The broader literature supports these findings across other insect taxa. Beetles, flies, and caterpillars exhibit similar scale-dependent attachment limitations when confronted with waxy, micro-rough, or pubescent plant surfaces [27,74]. Thus, the principles uncovered in ant–stem interactions likely reflect generalizable mechanisms applicable across arthropods.

## 5. Ecological and Evolutionary Implications

### 5.1. Ant Exclusion, Mutualisms, and Ecological Trade-Offs

While ants can function as defenders, they may also protect herbivores or disrupt pollination. Mechanical exclusion via stem surface traits can allow plants to selectively regulate ant access without relying on chemical deterrents. Such mechanical barriers represent low-cost, durable defense strategies that minimize energetic investment and reduce unintended ecological consequences [69,88,89].

**Figure 5:**
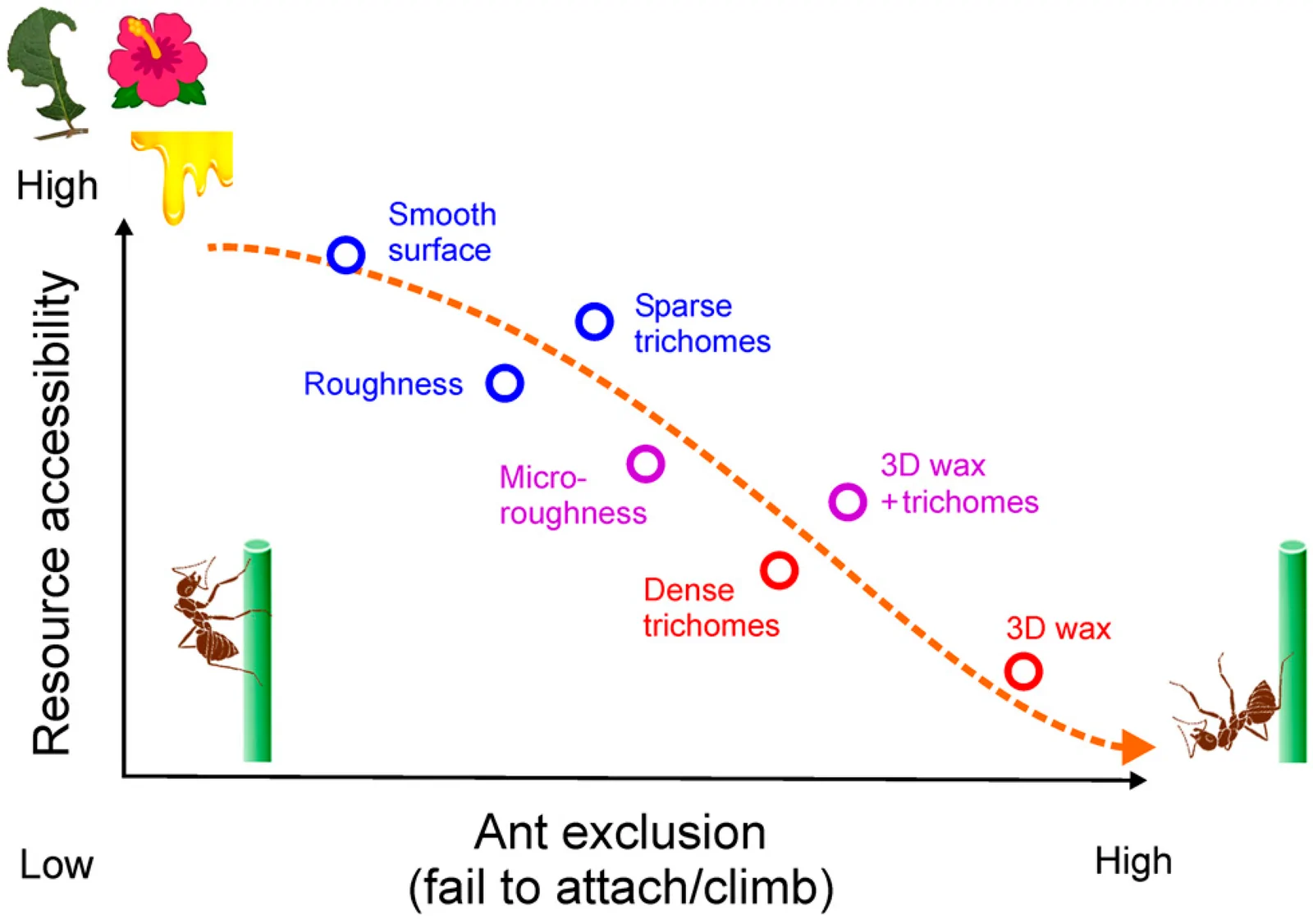
Conceptual image of the evolutionary trade-offs in plant surface traits. Illustration showing how multi-trait combinations (3D epicuticular wax, pubescence, and micro-roughness) can balance ant exclusion with resource accessibility, tuning ecological interactions. Each point represents a hypothetical plant strategy (not a quantitative result), with smooth surfaces allowing high resource access but low ant exclusion, 3D wax with moderate pubescence providing intermediate trade-offs, and 3D wax coverage or dense trichome-covered surfaces maximizing ant exclusion at the cost of reduced accessibility. Orange dashed line illustrates hypothetical evolutionary compromise between deterring herbivores and maintaining beneficial interactions.

The trade-off between accessibility and exclusion is evident in plants that rely on ant-mediated defense for certain tissues but must protect flowers or reproductive structures. Surface traits, such as waxy stems or dense pubescence, can exclude non-mutualistic ants (Figure 5) while allowing access to mutualists capable of navigating the microtopography [5,28]. This selective exclusion may be critical in shaping plant–ant co-evolutionary dynamics.

### 5.2. Evolutionary Diversification of Surface Traits

Comparative analyses indicate that stem surface traits are highly labile and may evolve rapidly in response to selective pressures from insect communities. Even closely related species (or even plant cultivars of the same species) can exhibit significant differences in wax coverage, trichome density, and epidermal cell geometry [25,29,30,43]. Such diversification likely reflects an adaptive response to local insect assemblages, resource distribution, and ecological context, consistent with the predictions of optimal defense theory and adaptive landscape models [90,91,92].

The interaction between multiple surface traits suggests that selection operates on trait combinations rather than individual features (Figure 3 and Figure 5). Multi-trait strategies, such as the combination of wax, pubescence, and (micro-)roughness, provide synergistic benefits and enhance mechanical filtering efficiency (Table 1). This integrative perspective helps to explain the high variability in plant surface microstructure observed across taxa and habitats.

### 5.3. Relevance to Managed and Agricultural Systems

In managed crop and horticultural systems, plant surface traits may have important consequences for pest exclusion, beneficial insect access, and crop protection. Experimental studies on crop plants have demonstrated that epicuticular waxes, surface roughness, trichomes, and epidermal architecture can alter insect attachment, locomotion, oviposition-site selection, and movement on leaves or stems, including in systems involving *Brassica*, *Pisum*, *Rumex*, *Vitis*, and other cultivated plants [43,52,73,75]. These findings demonstrate that surface-mediated effects are not restricted to wild plants and provide a mechanistic basis for considering plant surface architecture as a component of crop defense.

However, the extent to which these traits can be deliberately exploited in agriculture remains largely speculative. In particular, it is not yet established whether manipulating wax morphology, trichome density, or combinations of surface traits can reliably reduce pest pressure while maintaining access by pollinators and other beneficial insects. Field conditions may also modify these interactions through variation in humidity, rain, dust, microbial colonization, plant age, and natural insect communities. Consequently, future studies should test whether experimentally observed mechanical filtering persists under field conditions and whether it translates into measurable reductions in pest abundance or crop damage without compromising pollination or biological control. Such experiments, ideally using replicated field trials and multiple insect functional groups, will be necessary before plant surface architecture can be considered a practical component of integrated crop protection.

### 5.4. Taxonomic Scope and Generalizability

An important limitation of the current evidence base is that a substantial proportion of the experimental work on plant stem surfaces has been conducted with ants, and particularly with the generalist species *Lasius niger*. This species provides a useful model, because its attachment system and locomotory behavior are relatively well characterized and because it frequently encounters diverse plant surfaces. Nevertheless, *L. niger* should not be regarded as representative of all ants, or of insects in general. Formicids vary in body size, claw geometry, adhesive pad morphology, pad compliance, and locomotory behavior, and these differences may influence their responses to the same plant surface.

The evidence for broader generality therefore varies among mechanisms. Effects such as pad contamination by waxes, reduction of effective contact area, scale-dependent mismatch, and interference with claw interlocking have been demonstrated in several insect and arthropod groups, providing support for the underlying biomechanical principles [3,6,23,24,25,26,27,41,44,45,46,47,48,52,53,54,55,56,61,62,82,83,93,94]. However, the ecological consequences of these mechanisms, including reduced visitation or complete exclusion, cannot necessarily be extrapolated directly from one insect taxon to another. In particular, insects relying predominantly on either smooth adhesive pads, fibrillar pads, or claws may respond differently to the same combination of waxes, roughness, and trichomes.

We therefore regard mechanical filtering as a generalizable mechanistic framework rather than a universal response pattern. The framework predicts that the magnitude and direction of filtering depend on the compatibility between plant surface traits and the attachment and locomotory system of the insect. Testing this prediction will require comparative experiments using insects with contrasting attachment systems, body sizes, and ecological strategies. Such studies would provide a stronger basis for determining whether particular plant surface traits act as broad-spectrum filters or instead selectively affect particular functional groups of insects.

## 6. Methodological Advances

The reviewed studies exemplify a methodological tradition that integrates microscopy, surface characterization, behavioral assays, and attachment force measurements. Scanning electron microscopy provides detailed visualization of plant epidermal topography, wax morphology, and trichome structure and arrangement (e.g., [23,30,64,65,66,70]). Behavioral assays, including choice experiments and performance trials, quantify insect responses in controlled settings [23,24,28,29]. Force measurements of insect adhesion complement these approaches by providing quantitative links between surface structure and functional performance [4,87].

The broader literature demonstrates the value of these approaches in revealing generalizable patterns across taxa. Comparative studies across multiple plant species and insect groups allow for the identification of recurring biomechanical principles, including the importance of scale matching, multi-trait synergy, and contamination effects [23,26,47,55,70].

## 7. Bioinspired Perspectives

The mechanical principles underlying plant–insect interactions have potential relevance beyond their biological context. In particular, mechanisms such as reduction of effective contact area, pad contamination by surface materials, roughness-induced attachment mismatch, lubrication, and physical interference by surface protrusions are relevant to the design of surfaces with controlled adhesion and friction. Biological surfaces have consequently provided inspiration for slippery, self-cleaning, anti-adhesive, and antifouling materials [6,88,95,96].

However, translating these principles into engineered systems is not straightforward. The effectiveness of a particular mechanism depends on the properties of both the contacting organism/object and the surface, and combinations of mechanisms may produce outcomes different from those of individual surface traits. For example, hierarchical structures of plant waxes can reduce insect attachment through several simultaneously acting mechanisms, whereas engineered surfaces designed to reproduce only their topographic characteristics may not reproduce the same effect if surface chemistry, contamination, or lubrication differ. Similarly, trichome-like structures may interfere with insect locomotion through mechanical entanglement, but their implementation in technical surfaces requires consideration of durability, fouling, and manufacturing constraints.

The plant–insect interface therefore provides useful design principles rather than direct engineering templates. In particular, the concept of combining several surface characteristics to obtain selective effects may be relevant to future development of surfaces with controlled adhesion or organism–surface interactions. A systematic evaluation of how individual mechanisms and their combinations can be translated into materials engineering, pest management, and antifouling technologies represents an interesting direction for future work but is beyond the scope of the present perspective paper.

## 8. Conclusions

Plant stem surface microstructure represents a powerful, multifunctional mediator of plant–insect interactions. By integrating biomechanics, behavior, ecology, and evolutionary biology, the literature reviewed here establishes mechanical filtering as a unifying framework for understanding how plants regulate insect access (Figure 1). Multi-trait strategies involving epicuticular waxes, cuticular micro-roughness, pubescence, and epidermal geometry create synergistic effects that reduce unwanted insect visitation, while minimizing energetic costs. This synthesis provides a foundation for further comparative studies, experiments, and bioinspired applications.

## 9. Final Remarks

This perspective paper consolidates over a decade of research into a framework explaining how plant stem surface microstructure mediates plant–insect interactions. By highlighting the principles of mechanical filtering, scale-dependent attachment, and multi-trait synergy, we provide a unifying perspective that bridges biomechanics, ecology, and evolution. The synthesis also outlines avenues for future research, including comparative studies across plant and insect taxa, functional experiments manipulating multiple surface traits, and translation of plant microstructural principles and their combinations into bioinspired technologies.

## Figures and Tables

**Table 1:** Plant surface traits resulting in negative insect responses.

Surface Trait	Structural Characteristics	Insect Response	Representative Taxa	Key References
Smooth wax film/layer	Continuous hydrophobic layer	Low effect on ant friction and adhesion	Ants, flies	[24,86]
Epicuticular wax projections	Loose 3D wax layer	Slipping, pad contamination, reduced visitation	Ants, beetles	[6,24,25,28,29,88]
Micro-rough cuticle (incl. cuticular folds)	Submicron roughness	Claw and pad mismatch	Ants, beetles	[26,29,85]
Dense pubescence	High trichome density	Reduced contact area, locomotion impairment	Ants, caterpillars	[28,69]
Convex/papillate epidermal cells	Protuberant cell geometry	Reduced contact area, claw interlocking failure	Ants, beetles	[30,75]

## Data Availability

No new data were created or analyzed in this study. Data sharing is not applicable to this article.
